# Dietary 25(OH)D_3_ supplementation to gestating and lactating sows and their progeny affects growth performance, carcass characteristics, blood profiles and myogenic regulatory factor-related gene expression in wean-finish pigs

**DOI:** 10.5713/ab.21.0304

**Published:** 2021-10-29

**Authors:** Santi Devi Upadhaya, Thau Kiong Chung, Yeon Jae Jung, In Ho Kim

**Affiliations:** 1 Department of Animal Resource and Science, Dankook University, Cheonan 31116, Korea; 2 DSM Nutritional Products Asia Pacific, Mapletree Business City, Singapore 117440

**Keywords:** Growing Pigs, Hydroxy Vitamin D, Myogenesis, Pork Meat Quality, Sows

## Abstract

**Objective:**

This experiment investigated the effects of supplementing vitamin D_3_-fortified sow and progeny diets with 25(OH)D_3_ on growth performance, carcass characteristics, immunity, and pork meat quality.

**Methods:**

The present study involved the assessment of supplementing the diet of sows and their progeny with or without 25 (OH)D_3_ in a 2×2 factorial arrangement on the performance and production characteristics of wean-finish pigs. Forty-eight multiparous sows were assigned to a basal diet containing 2000 IU/kg vitamin D_3_ and supplemented without (CON) or with (TRT) 50 μg/kg 25 (OH)D_3_. At weaning, a total of 80 pigs each from CON and TRT sows were allocated to weaning and growing-finishing basal diets fortified with 2,500 and 1,750 IU/kg vitamin D_3_ respectively and supplemented without or with 50 μg/kg 25(OH)D_3_.

**Results:**

Sows fed 25(OH)D_3_-supplemented diets improved pre-weaning growth rate of nursing piglets. A significant sow and pig weaning diet effect was observed for growth rate and feed efficiency (p<0.05) during days 1 to 42 post-weaning. Pigs consuming 25(OH)D_3_-supplemented diets gained weight faster (p = 0.016), ate more (p = 0.044) and tended to convert feed to gain more efficiently (p = 0.088) than those fed CON diet between days 98 and 140 post-weaning. Supplemental 25(OH)D_3_ improved water holding capacity and reduced drip loss of pork meat, increased serum 25(OH)D_3_ level, produced higher interleukin-1 and lower interleukin-6 concentrations in blood circulation, downregulated myostatin (*MSTN*) and upregulated myogenic differentiation (*MYOD*) and myogenic factor 5 (*MYF5*) gene expressions (p<0.05).

**Conclusion:**

Supplementing vitamin D_3_-fortified sow and wean-finish pig diets with 50 μg/kg 25(OH)D_3_ significantly improved production performance suggesting their current dietary vitamin D_3_ levels are insufficient. In fulfilling the total need for vitamin D, it is strongly recommended to add 50 μg/kg 25(OH)D_3_ “on top” to practical vitamin D_3_-fortified sow and wean-finish pig diets deployed under commercial conditions.

## INTRODUCTION

Vitamin D is an important micronutrient which is necessary for the growth and maintenance of functional skeleton and helps to sustain health and improve longevity. 25(OH)D_3_ is a form of vitamin D_3_ that has been used in animal diets as a new source of vitamin D [1,2] because it is an intermediary metabolite that bypasses the liver metabolism and is readily available to animals. Moreover, it is five times more potent in raising vitamin D status of humans than an equivalent amount of vitamin D_3_ [3]. Increases in piglet body weight (BW) at birth and weaning were observed when dams were supplemented 2,000 IU/kg 25(OH)D_3_ compared to piglets from dams supplemented 2,000 IU/kg vitamin D_3_ [4]. Thayer et al [5] demonstrated that the feed efficiency of progeny was improved in the nursery from day 0 to 59 when the dams and nursery pigs were fed diets containing 1,500 IU/kg vitamin D_3_+50 μg/kg 25(OH)D_3_ compared with pigs from dams fed diets containing 500 IU/kg vitamin D_3_+ 25 μg/kg 25(OH)D_3_ but no differences in the growth performance of pigs in the finisher phases were observed. A previous study by Flohr et al [6] noted that growing pigs from sows fed 50 μg/kg 25(OH)D_3_ achieved higher average daily gain (ADG) than those pigs from sows fed 800 IU/kg vitamin D_3_. Duffy et al [7] demonstrated that pigs offered 25(OH)D_3_ diets exhibited highest serum 25(OH)D_3_ concentration and subsequently exhibited the highest *Longissimus thoracis* total vitamin D activity. However, other studies reported that finishing growth was unaffected by the supplementation of vitamin D_3_ alone or in combination with 25(OH)D_3_ [8–10].

The benefits of vitamin D go beyond the function of the regulation of calcium and phosphorus homeostasis as it influences bone development, growth performance, immune status and production. Vitamin D metabolites control the expression of more than 200 genes through activation of the vitamin D receptor, which regulates or modulates gene expression within the target cell [11]. This gives vitamin D a role in many functions in swine, including immunity, muscle function, and reproduction. The development of fetal muscle has far-reaching consequences for overall growth performance and health. In agricultural research, the importance of myostatin (*MSTN*) and myogenic regulatory factors expression in early stages of development is well understood to impact meat quality and ultimate meat yield. Zhou et al [10] demonstrated that supplementation with 25(OH)D_3_ to dam’s diet can promote prenatal and postnatal skeletal muscle development of pig offspring by modulating the expressions of muscle transcription factors. A population-based mother-offspring cohort study in humans suggested that maternal vitamin D status during late pregnancy might influence muscle strength of offspring at 4 years of age [12]. Thus, vitamin D supplementation has been demonstrated to exert a range of effects on the development of skeletal muscle of humans and animals [10,13,14]. An alteration in fetal muscle characteristics was observed in fetuses from gilts fed the 25(OH)D_3_ compared to fetuses from gilts fed vitamin D_3_ [15] when fed at concentrations above the basal requirement estimate.

Studies on how the performance and production characteristics of finishing pigs might be affected by supplementation of the dam’s diet with 25(OH)D_3_ followed by supplementation to their progeny diets throughout the wean to finish period are very limited but based on some of the human literature, it could have important scientific and commercial implications. Therefore, the objectives of the experiment herein were to: i) evaluate the performance and production characteristics of pigs fed dietary supplementation of 50 μg/kg 25(OH)D_3_ (equivalent to 2,000 IU/kg vitamin D_3_) during wean-to-finish period, ii) evaluate the influence of maternal 25(OH)D_3_ supplementation on the performance and production characteristics, blood metabolites, carcass characteristics and myostatin/myogenic regulatory factor gene expression of finishing pigs. We hypothesized that supplementation of the sow diets with 50 μg/kg 25(OH)D_3_ would improve reproduction and pre-weaning performance and that the effects would extend to post-weaning performance, muscle gene expression and meat quality. We also hypothesized that these maternal effects would be enhanced by 25(OH)D_3_ supplementation of the diets offered to the progeny after weaning.

## MATERIALS AND METHODS

### Animal care

The experimental protocol (DK-2-1613) describing the management and care of animals were reviewed and approved by the Animal Care and Use Committee of Dankook University, Cheonan, South Korea.

### Source of tested product

The tested product, 25(OH)D_3_, is the active ingredient of ROVIMIX HyD 1.25% (DSM Nutritional Products Ltd., Kaiseraugst, Switzerland). ROVIMIX HyD 1.25% was added to the basal diet via a premix of 500 g/tonne (i.e., 0.05% of diet) containing 50 mg of 25(OH)D_3_ by replacing the same amount of corn.

### Experimental design, animals, and diets

A total of 48 multiparous sows (parity 3+) ([Yorkshire× Landrace]×Duroc) were assigned to either basal diet fortified with 2,000 IU/kg vitamin D_3_ as the control (CON; 24 sows) or the control diet supplemented with 50 μg/kg 25(OH)D_3_ (TRT; 24 sows) based on their BW and expected farrowing date. Sows were offered a gestation diet at 2.5 kg daily in 2 equal meals during the gestation period. The gestation and lactation diets of sows were formulated to meet or exceed the nutrient requirements of pigs as recommended by National Research Council [16]. Approximately 8 to 9 days before farrowing sows were shifted to farrowing crates and fed gestation diet. After day 1 of farrowing sows were fed a lactation diet which was gradually increased from 2.5 kg/d to *ad libitum* feed access by day 5 of lactation. Newly born piglets were weighed after farrowing. Litter size at birth per sow and mortality were recorded. Piglets were weaned at an average of 21 days of age.

A total of 160 weaned piglets were randomly selected for the assessment of growth performance, blood profiles, meat quality and muscle gene expression during wean-finish period. Each half of the piglets (80 each) born to sows fed either CON or TRT diet were randomly assigned again to diets that consisted of basal diet fortified with 2,500 IU/kg or 1,750 IU/kg vitamin D_3_ (for weaning and growing-finishing respectively) as control (CON) or the control diet supplemented with 50 μg/kg 25(OH)D_3_ (TRT). Assignment across treatments was based on their litter, age, and weaning weight in a 2×2 factorial design. There were 8 replicate pens for each sow-wean to finish treatment combination with five pigs (2 barrows and 3 gilts) per pen for the four sow and post weaning combinations. Pigs from the same litter were assigned to different pens for proper randomization. Pigs were fed experimental weaning diets for 42 days in three phases; phase 1 (day 1 to 7); phase 2 (day 8 to 21) and phase 3 (day 22 to 42). After day 42, piglets were offered experimental growing diets in 2 phases (day 43 to 70 and day 71 to 98) and finishing diets (day 98 to 140). Each pen was equipped with a one-sided self-feeder and a nipple drinker to enable *ad libitum* feed and water intake. The composition of control diets for sows, weaned piglets and grower-finish pigs presented in Table 1, 2 and 3, respectively. Progeny diets were formulated to meet or exceed the nutrient requirements of pigs [16] based on expected weight ranges (7 to 110 kg) for each growth phase investigated.

### Sampling and measurements

Feed samples (in duplicates) including sows gestation and lactation diets, progeny weaning (phase 1, 2, and 3 diets), growing (phase 1, and 2 diets) and finishing diet were analyzed for crude fat (method 954.02), ash (method 942.05), acid detergent fiber (method 973.18), amino acids (method 982.30E), calcium (method 984.01) and phosphorous (method 965.17) following the procedures established by the Association of Official Analytical Chemists (AOAC) [17]. The neutral detergent fiber assayed with heat stable amylase was determined using the method of Van Soest et al [18]. The gross energy was determined using bomb calorimeter (Mode 1241; Parr Instrument Co., Moline, IL, USA).

### Growth performance

The individual BW of weaned piglets was recorded at days 1, 42, 98, and 140. Feed intake of each pen was recorded every 2 weeks. The feed consumption and individual BW were used to calculate the ADG, average daily feed intake (ADFI), and gain-to-feed ratio (G:F).

### Blood profile

Eight pigs per treatment were randomly selected (1 pig per pen) and bled via jugular venipuncture at the end of days 42, 98, and 140. Blood samples (5 mL) were collected from same pigs at different collection times into vacuum tubes (containing no additive) to obtain serum. Serum was separated by centrifugation for 15 min at 3,000×g at 4°C and stored at 4°C until determination for serum immunoglobulin G (IgG), using Automatic Biochemical Blood Analyzer (HITACHI 747, Tokyo, Japan). Serum pro-inflammatory cytokines such as interleukin-1 (IL-1), tumor necrosis factor-alpha (TNF-α), interleukin-6 (IL-6) and tumor necrosis factor beta (TNF-β) were measured in blood using an ELISA kit (R & D Systems, Minneapolis, MN, USA). Serum 25-OH-D_3_ levels were determined by electro chemiluminescence immunoassay (ECLIA) (Roche Diagnostics, Mannheim, Germany).

### Backfat thickness

During the wean to finish period, backfat thickness was measured on all pigs at days 70, 98, and 140. Backfat thickness was measured 5 cm from the right-hand side of the mid line from three different sites (shoulder, mid-back and loin at a position directly above the point of elbow, last rib and last lumbar vertebra) using a real-time ultrasound instrument (Piglog 105, SFK Technology, Herlev, Denmark) as described by Kim et al [19].

### Meat quality

At the end of the experiment all pigs were transferred to commercial slaughterhouse and slaughtered. Then 8 pigs per treatment (1 pig per pen each treatment) were randomly selected based on live weight for evaluating meat quality. The carcasses from the selected animals were placed in a conventional chiller at 4°C. After a 24 h chilling period, carcasses were fabricated into primal cuts. Meat samples including lean, and fat were taken via perpendicular cut loins into 2 cm thick chops beginning from the 10th and 11th ribs region. The pH of muscle was measured 24 h after postmortem using a pH meter (Testo 205, Testo, Lenzkirch, Germany). Sensory evaluation was conducted by six trained panelists to evaluate the color darkness, firmness and marbling of fresh loin samples using a five-point assessment scheme according to the procedures established by the National Pork Producers Council [20]. Immediately after the subjective tests were conducted, meat color of the *longissimus* muscle (LM) as lightness (L*), redness (a*), and yellowness (b*), was determined using a Minolta Chromameter (CR-210, Minolta, Tokyo, Japan) to evaluate the freshly cut surface after 30 min of blooming at 4°C. Water-holding capacity (WHC) was measured using methods of Kauffman et al [21]. Briefly, a 0.2 g sample was pressed at 20,684 kPa for 3 min onto laboratory grade 125-mm-diameter filter paper. The areas of pressed sample and expressed moisture were delineated and determined with a digitising area-line sensor (MT-10S; M. T. Precision Co. Ltd, 123 Tokyo, Japan). A ratio of water area: meat area was calculated to give a measure of WHC, with smaller ratio indicating higher WHC. The LM area was measured by tracing the LM surface at 10th rib, which also used the above-mentioned digitizing area-line sensor. Cook loss was determined as described previously by Sullivan et al [22]. Briefly, 5 g of meat sample were heat-treated in plastic bags separately in a water bath (100°C) for 5 min. Samples were cooled at room temperature. Cooking loss was calculated as (sample weight before cooking-sample weight after cooking)/sample weight before cooking×100. Drip loss was measured using ~2 g of meat sample according to the plastic bag method. Briefly, two (2.5 cm) chops were weighed placed in a drip loss tube (C. Christensen Laboratory, Hillerod, Denmark), and held at 2°C for 24 h. Then, meat samples were removed, blotted dry on paper towels, and re-weighed. Differences between sample weights were used to calculate drip loss percentage.

### Determination of relative mRNA expression of gene encoding for myostatin/myogenic regulator factor genes using real-time polymerase chain reaction

For the determination of the relative mRNA expression encoding for myostatin/myogenic regulator factor genes (*MSTN*, myogenic differentiation [*MYOD*], myogenin [*MYOG*], and myogenic factor 5 [*MYF5*]), quantitative reverse-transcription polymerase chain reaction (qRT-PCR) was done. One μg RNA was extracted from muscle tissues of randomly selected 8 pigs per treatment at day 140 and was used for complementary DNA synthesis with a Maxima First-strand cDNA Synthesis Kit (Life Technologies, Carlsbad, CA, USA). The primers for qRT-PCR for each gene transcript were designed using Primer3 (http://frodo.wi.mit.edu/; Table 4). The qRT-PCR was performed using a 7500 Fast Real-time PCR System (Applied Biosystems, Foster City, CA, USA) with the following conditions: 94°C for 3 min, followed by 40 cycles at 94°C for 30 s, 59°C to 61°C for 30 s, and 72°C for 30 s. Melting curve profiles were analyzed for the amplicons. qRT-PCR data were normalized to the expression of glyceraldehyde 3-phosphate dehydrogenase (GAPDH), an endogenous control gene, and calculated using the 2−ΔΔCt method, where ΔΔCt (cycle threshold) = ΔCt (treated) − ΔCt (control) and ΔCt = Ct of the target gene − Ct of *GAPDH* (treated or control, respectively) as described by Livak and Schmittgen [23].

### Statistical analysis

The present study involved the assessment of supplementing the sow and progeny diet with 25(OH)D_3_ in a 2×2 factorial arrangement. Two-way analysis of variance (ANOVA) was used to assess the effects of supplementing the sows’ basal diets (fortified with 2,000 IU/kg vitamin D_3_) with or without 50 μg/kg 25(OH)D_3_ during gestation to lactation and weaning-finish pigs’ basal diets (fortified with 2,500 IU/kg or 1,750 IU/kg vitamin D_3_ respectively) supplemented with or without 50 μg/kg of 25(OH)D_3_. For growth performance indices (BW, ADG, ADFI, and G:F) at different time points, data were also analyzed using a repeated measure mixed model with initial value at the start of the experiment as a covariate (SAS Inst. Stat. v.9.3, Cary, NC, USA) [24]. The interactive effects between sow and their progeny diets were also determined. Variability in the data was expressed as standard error of means and probability level of p<0.05 was considered significant and p<0.10 as trends.

## RESULTS

### Pre-weaning piglet growth

The effect of 25(OH)D_3_ supplementation to sow diet on pre-weaning piglet growth is presented in Table 5. Litter size at birth was not significantly affected by treatment but the number born alive was higher (p<0.05) for 25(OH)D_3_-fed sows. The weaning weight and pre-weaning ADG of piglets born to sows from the 25(OH)D_3_ group were also higher (p<0.05) than the control group.

### Growth performance post weaning

The results for the growth performance from wean-to-finish period are presented in Table 6. The BW was not affected at 1, 42, 98, and 140 days post weaning in pigs raised from dams that received the supplementation of 25(OH)D_3_ in their gestation and lactation diets compared with their control counterparts. However, 25(OH)D_3_ supplementation of the progeny diets offered after weaning tended (p = 0.07) to increase BW at day 42 and significantly increased (p< 0.05) BW at days 98, and 140. There were significant time effects for growth performance indices in wean-finish pigs with 25(OH)D_3_ supplementation such that the measured mean values at day 42, 98, and 140 were greater (p<0.0001) than initial mean values.

For the nursery period after weaning (days 1 to 42), ADG and G:F were higher (p<0.05) in piglets born to sows receiving 25(OH)D_3_ supplemented diets during gestation and lactation. In addition, 25(OH)D_3_ supplementation of the piglet diet increased (p<0.05) ADG and G:F during the same period (days 1 to 42). For the growing period (days 42 to 98), performance was not affected by treatment. For the finish period (days 98 to 140), pigs offered the supplemented diets grew significantly faster and tended to eat more (p = 0.069) than their control counterparts. 25(OH)D_3_ supplementation to the diet offered pigs after weaning also significantly increased ADG and ADFI during the overall experimental period. There were no interactive sow and piglet diet effects on growth performance indices during the wean-finish period.

### Blood profile

The effect of 25(OH)D_3_ supplementation on blood metabolites is presented in Table 7. There was no significant sow diet 25(OH)D_3_ supplementation effect on blood profiles of weaned piglets. However, a significant increase (p<0.05) in serum 25(OH)D_3_ concentration during days 42, 98, and 140 was observed for pigs receiving the 25(OH)D_3_ supplemented diets in the wean to-finish period. In addition, lower IL-6 (p< 0.05) during day 42, higher P concentration during day 98 and a higher (p<0.05) IL-1 concentration during day 140 was observed in pigs fed 25(OH)D_3_ supplemented diets during wean-to-finish period compared with those receiving the control diet. The supplementation of 25(OH)D_3_ either in sow diet during gestation and lactation or piglet diet during wean-finish period did not have significant effects on serum IgG, TNF- α and TNF-β concentrations.

### Backfat thickness and meat quality

The effect of 25(OH)D_3_ supplementation on backfat thickness and meat quality is presented in Table 8. A trend in increased backfat thickness at days 70 (p = 0.057) and 140 (p = 0.074) was observed in pigs born to sows fed 25(OH)D_3_ supplemented diets compared with those fed the CON diets. However, no significant effects on backfat thickness were observed for pigs fed 25(OH)D_3_ during wean-finish period compared with those fed control diet.

The sensory evaluation, and meat color, remained unaffected in pigs receiving 25(OH)D_3_ supplemented diet during wean-to-finish period as well as from sow diet compared with control. However, drip loss was reduced (p<0.05) during days 5 and 7 of post mortem storage and WHC was increased (p< 0.05) and pH was lower (p<0.05) in pigs receiving 25(OH)D_3_ from dietary supplementation during wean-finish period. Supplementation of the sow diets significantly reduced (p< 0.05) drip loss at day 7 of post mortem storage and tended to increase (p = 0.057) muscle pH. There were no interactive sow and piglet diet effects on backfat thickness and meat quality.

### Expression of mRNA encoding for myostatin/myogenic regulatory factors

The effect of 25(OH)D_3_ supplementation on the expression of *mRNA* encoding for myostatin/myogenic regulatory factors is presented in Table 9. The *MSTN* gene was significantly down-regulated (p<0.05) whereas *MYOD* and *MYF5* genes (p<0.05) were upregulated for pigs born to sows receiving 25(OH)D_3_ supplemented diets during gestation and lactation. In addition, the *MSTN* gene expression was significantly down-regulated (p<0.05) whereas *MYF5* (p<0.05) was upregulated for pigs receiving 25(OH)D_3_ There were no sow or piglet diets 25(OH)D_3_ supplementation supplemented diet post weaning. There were no sow or piglet diets 25(OH)D_3_ supplementation effects observed for *MYOG* gene expression. There were no interactive effects on any of the measured parameters.

## DISCUSSION

The massive change in growth potential and management of commercial pigs has initiated a new concept of optimum vitamin nutrition so as to supply appropriate levels of vitamins during specific physiological phases of animals leading to positive results that go beyond the initial objective of preventing deficiency. To meet the body needs during different phases of growth, the vitamin D_3_ dose recommended by DSM Nutritional Products Limited [25] (above National Research Council [16] recommendation levels) was fortified in the premix of the basal diets in the present study and considered as the control diet.

There is also increased interest in understanding how maternal nutrient supplement can impact progeny growth and health. A study by Hines et al [15] concluded that the increase in fetal muscle development was due to the increase in maternal 25(OH)D_3_. The main purpose of the current study was to evaluate the supplementation of 50 μg/kg 25(OH)D_3_ to the basal diets of sows and their progeny on the growth performance, blood profile, muscle gene expression and production of wean to finish pigs.

In the current study, 25(OH)D_3_ supplementation of the diets fed during gestation and lactation improved pre-weaning growth rate and growth and feed efficiency in the period to day 42 post weaning. Weber et al [4] noted that the weaning weight of pigs born to sows fed 50 μg/kg 25(OH)D_3_ was higher than those fed 2,000 IU/kg vitamin D_3_, indicating the source of vitamin D was influential in improving the growth of pigs. In addition, Zhou et al [26] reported that feeding a combined supplement of the two vitamin D_3_ sources (50 μg/kg each 25(OH)D_3_ and vitamin D_3_) during gestation and lactation increased piglet growth during the first 2 weeks of lactation. In the study reported herein, the sow diet 25(OH)D_3_ supplementation had no effect on growth performance after 42 days post weaning but supplementation of 50 μg/kg 25(OH)D_3_ to the basal diets offered post weaning led to heavier BW at days 42, 98, and 140 and a higher ADG from weaning to day 42, between days 98 and 140 and over the entire wean to finish period. Tousignant et al [27], reported that the oral administration of vitamin D_3_ to suckling pigs resulted in higher BW at weaning and 7 days post weaning but weights did not differ at 26 days post weaning compared with control. However, several other reports indicated that nursery and finishing growth was unaffected by the supplementation of vitamin D_3_ alone or in combination with 25(OH)D_3_ [8–10]. The difference in outcomes between our study and these others may be due to the doses of vitamin D tested. For example, Thayer et al [5], investigated total vitamin D (D_3_ alone or combined with 25(OH)D_3_) levels per kg diet ranging from 1,500 to 3,500 IU, 1,000 to 2,000 IU, and 800 to 1,600 IU for sow, nursery pigs, and growing and finishing pigs respectively and reported no effects of treatments on reproduction or pig growth performance. In the current study the total vitamin D/kg diet (basal D_3_ alone or basal D_3_ plus 2,000 IU 25(OH)D_3_) ranged from 2,000 to 4,000 IU, 2,500 to 4,500 IU, and 1,750 to 3,750 IU for sows, nursery and growing and finishing pigs, respectively. It’s probable that that contemporary pigs may need higher dietary vitamin D levels to maximize their lifetime performance than previously thought [28]. The interplay between vitamin D and the growth hormone (GH)/insulin-like growth factor (IGF)-1 system is not fully understood. Insulin-like growth factor-1 is produced by the liver in response to GH stimulation. Both GH and IGFs form part of the somatropic axis, which promotes whole body growth and development via action on key metabolic organs including the liver, skeletal muscle and bone. Growth hormone directly regulates renal 1α-hydroxylase (catalyzed the conversion of 25(OH)D_3_ to 1α,25 (OH)_2_D_3_ and therefore modulates vitamin D metabolism mediated by IGF-1 [29]. It has been suggested that the supplementation of vitamin D to the diet of humans with vitamin D deficiency served as a link between the proliferating cartilage cells of the growth plate and GH/IGF-1 secretion and the increase in IGF-1 and 25(OH)D_3_ levels. The improvement in growth of pigs receiving 25(OH)D_3_ either from the sows or through their nursery-finish diets might partly be due to the activation of the GH/IGF-1 axis.

Supplementation of D_3_ or 25(OH)D_3_ results in increased concentrations of Ca^2+^ in the blood and muscle tissue [30,31]. Higher level of calcium in muscles activates calpain proteinase system (CPS), which consists of μ-calpain and m-calpain activated by Ca^2+^ ions and endogenous calpain inhibitor-calpastatin. In addition to playing a role in post-mortem meat tenderness, CPS also regulates skeletal muscle growth [32]. In our study, the activity of CPS was not measured, but it was highly likely that CPS was activated when feeding D_3_ plus 25(OH)D_3_ from wean-finish and was involved in regulating muscle growth, resulting in increased growth performance as evidenced in the current study.

Several researchers have suggested the role of vitamin D_3_ and its active metabolite 1α, 25(OH)_2_D_3_ in modulating immune response [33,34]. In a human study, it was suggested that there is a direct link between 25(OH)D_3_ and IgG [35]. Vitamin D also has an effect on the inflammatory profile of monocytes by down-regulating the expression and production of several pro-inflammatory cytokines including TNF-α, IL-1β, IL-6, and IL-8 [36,37]. In the present study, only IL-6 and IL-1 were affected by 25(OH)D_3_ supplementation of the diets offered post weaning. The former was reduced at 42 days and the latter increased at 140 days suggesting positive effects of 25(OH)D_3_ on some immune related markers. The longer-term consequences on animal health remain to be established. Several studies reported that supplementation of 25(OH)D_3_ resulted in increased serum 25(OH)D_3_ response in sows, neonatal pigs, nursery pig, grower, and finisher pigs [4,5,7,38–40]. In the present study, an increase in serum 25(OH)D_3_ and Ca concentration was detected in growing pigs that received diets supplemented with 50 μg/kg 25(OH)D_3_ post weaning. Conversely no significant sow diet effects were observed for these parameters in pigs. The lack of sow diet effect on serum 25(OH)D_3_ concentration of their progeny during the nursery and grower-finisher period is unclear but this may have differed if it was measured at weaning.

Backfat thickness is one of the traits that have an important influence on the profitability of swine industry. It is used as a tool to figure out the dietary requirements for the optimization of growth as well as to determine the price [41]. In the present study, the backfat thickness of growing-finish pigs on days 70 and 140 tended to be higher for pigs raised from sows fed the 25(OH)D_3_ supplemented diets. The increase in backfat thickness could be in part due to the higher growth rate of the 25(OH)D_3_ group in the nursery phase. Otherwise the small increase in fat thickness is difficult to explain as the sow treatments had minor effects on pig performance after day 42 post weaning.

Vitamin D may play an influential role in enhancing pork quality [7,42]. In a recent study, Duffy et al [7] demonstrated that pigs offered 25(OH)D_3_ diets exhibited highest serum 25(OH)D_3_ concentration and subsequently exhibited the highest *Longissimus thoracis* total vitamin D activity while Wilborn et al [43] demonstrated that supplementation of 2,000 IU/kg vitamin D_3_ to finishing pigs did not show detectable effects on a* or b* values but resulted in lower L* values compared to the meat evaluated from pigs fed control diets. While Wiegand et al [44] demonstrated that the supplementation of 500,000 IU of vitamin D_3_ for 3 days lowered L* values, increased a* values and did not affect b* values at 7 and 14 days post-mortem compared to control animals. In the present study, the sensory evaluation of meat, meat color, cooking loss, and LM area were unaffected by supplementation of 50 μg/kg 25(OH)D_3_ during the wean-to-finish period or through maternal transfer. The drip loss was reduced after 5 and 7 days of storage by supplementation of the sow and post weaning diets with 25(OH)D_3_ with the latter having the greater effect. Other aspects of meat quality affected by treatment included WHC and muscle pH. The former was enhanced in the meat from pigs offered the supplemented diets after weaning and the latter reduced by the same diets but increased in the muscle of pigs born to sows fed the supplemented diets.

The study by Wilborn et al [43] also reported a trend in reduction in drip loss after 8 days of storage in the meat obtained from pigs supplemented with high levels (40,000 or 80,000 IU) of vitamin D_3_. The variation in findings with regards to meat quality among different studies may be due to the difference in the sources of vitamin D used, treatment duration and dose of vitamin D. Interestingly, supplementation of the sow diets and progeny diets with 25(OH)D_3_ had opposite effects on muscle pH. The former increased and the latter reduced it. Though pork tenderness was not assessed in this study, calcium ions can improve tenderness when they are introduced into meat by injection [45] and infusion [46]. Vitamin D and 25(OH)D_3_ naturally increase serum and muscle Ca levels [30] and activates both μ-calpain and m-calpain and their inhibitor calpastatin, improving pork meat tenderness [32]. Dietary supplementation of pigs with 25(OH)D_3_ may be an effective strategy to increase pork meat tenderness without generating concerns of high vitamin D3 residues in meat. Unpublished field data by our group (T. K. Chung, personal communication, June 8, 2020) showed that feeding pigs with 25(OH)D_3_ dosed at 50 μg per kg diet in the presence of dietary vitamin D_3_ from wean to finish produced fresh pork with significantly lower Warner Bratzler Forces and cooked pork with significantly better tenderness scores assessed by trained pork meat panelists. Tenderness is a major driver of consumer perceptions of pork eating quality and the role of vitamin D in improving it warrants further research.

In the last few decades, a growing number of studies concerning the muscular effects of vitamin D supplementation and research on the vitamin D receptor in muscle cells have contributed to understanding the role and actions of vitamin D in muscle tissue and on physical performance. Vitamin D and its receptor are important for normal skeletal muscle development and in optimizing muscle strength and performance [47]. A study by Olsson et al [48] indicated the expression of vitamin D receptor at the cellular level and noted there is a direct effect of vitamin D on human skeletal muscle precursor cells. In another study, mice lacking vitamin D receptor showed the skeletal muscle phenotype having smaller and variable muscle fibers and immature muscle gene expression that persisted even during the adult age suggested the role of vitamin D in muscle development [49,50]. In a study by Garcia et al [51], it was demonstrated that inclusion of 25(OH)D_3_ to C_2_C_l2_ skeletal muscle cells induced an expression of several myogenic markers such as *MYOD*, myogenin at different stages of differentiation, and reduced the expression of *MSTN*, which is the negative regulator of muscle mass. In the present study, we also evaluated the expression of several genes that regulate muscle growth and differentiation. The inclusion of 50 μg/kg 25(OH)D_3_ in the sow and progeny diets reduced the expression of *MSTN* gene in finishing pig. It has been reported that the *MSTN* gene is the negative regulator of muscle mass [52,53].

Previous studies suggested that there is a direct effect of 25(OH)D_3_ on increasing the expression of follistatin during muscle cell differentiation which antagonizes *MSTN* by a direct protein interaction, preventing the inhibitory effects of *MSTN* [51,54,55]. Increased expression of the pro-myogenic skeletal markers *MYOD* and *MYF5* was observed for finishing pigs from sows fed 25(OH)D_3_ supplemented diets in gestation and lactation. Expression of *MYF5* but not *MYOD* was also enhanced in finishing pigs receiving 25(OH)D_3_ supplemented diets after weaning. However, no effect on *MYOG* gene expression was seen which agrees with the results of Braga et al [56] to some extent who indicated the downregulation of *MSTN* gene and upregulation of *MYOD*, *MYOG* in satellite cells from 8-week old C57/BL6 mice incubated with 1α,25(OH)_2_D_3_. The results show that, 25(OH)D_3_ supplementation in sow diets exerted a long-term effect on muscle gene expression with the changes indicative of enhanced muscle development. The results need to be confirmed and the implications on animal performance and carcass traits remain to be elucidated.

## CONCLUSION

The findings of the current study demonstrate unequivocally that the addition of 50 μg/kg 25(OH)D_3_ (a metabolite of vitamin D_3_) to gestation and lactation basal diets fortified with vitamin D_3_ enhanced ADG and G:F of progeny during day 1 to 42, reduced drip loss during day 7 of meat sample storage. Pigs offered diets containing 25(OH)D_3_ after weaning were heavier at days 98 and 140 and had higher daily gain between days 99 and 140 than those offered the control diets. Overall pigs offered the 25(OH)D_3_ supplemented diets had higher ADG, feed intake, were feed efficient, and had improved water holding capacity in meat samples than pigs fed basal diets without 25(OH)D_3_ supplementation. The serum 25(OH)D_3_ concentration during days 42, 98 and 140 was higher for pigs receiving the 25(OH)D_3_ supplemented diets in the wean to-finish period. In addition, lower IL-6 and a higher IL-1 concentration was observed in pigs fed 25(OH)D_3_ supplemented diets during wean-to-finish period suggesting the positive effects of 25(OH)D_3_ on the health of pigs. The myogenic markers such as *MYOD* was upregulated and *MSTN* gene expression which is negative regulator of muscle mass was downregulated by the inclusion of sows and their progeny post-weaning diets with 50 μg/kg 25(OH)D_3_ suggesting its role in improving muscle tissue. These findings indicate that the current vitamin D_3_ recommendation for sow and wean-finish pigs is insufficient and that including 25(OH)D_3_ at the dosage of 50 μg/kg to their vitamin D_3_-fortified basal diets would on one hand fulfill their total needs for vitamin D and on the other hand improve their production and performance traits.

## Figures and Tables

**Table 1:** Ingredient composition of sow basal diets (as-fed basis, %)

Items	Gestation	Lactation
Corn	42.52	33.01
Wheat	22.0	23.0
Rice bran	-	2.0
Wheat bran	10.0	8.31
Soybean hull	6.2	-
Palm kernell meal	2.0	-
Soybean meal	3.0	6.0
Dehulled soybean meal	5.94	12.96
Coconut powder	-	1.0
Soybean oil	1.52	3.74
Molasses	3.0	2.0
Bakery by product	-	4.0
Limestone	1.18	1.23
Mono-calcium phosphate	0.74	0.68
Salt	0.50	0.50
DL-Methionine (98%)	0.02	-
L-Threonine (98%)	0.09	0.05
L-Lysine HCl (25%)	0.59	0.6
Choline chloride (50%)	0.15	0.12
Vitamin/mineral mixture^1)^	0.55	0.80
Calculated composition		
Digestible energy (MJ/kg)	13.65	14.82
Metabolizable energy (MJ/kg)	12.64	13.61
Analyzed composition (%)		
Crude protein	13.0	16.3
Crude fat	3.8	6.7
Crude ash	4.9	5.0
ADF	4.1	3.3
aNDF	16.6	10.4
Lysine	0.68	0.93
Methionine	0.19	0.24
Threonine	0.48	0.56
Calcium	0.75	0.75
Phosphorus	0.50	0.54

ADF, acid detergent fiber; aNDF, neutral detergent fiber assayed with heat stable amylase.

1) Vitamins provided per kilogram of complete diet: vitamin A, 10,000 IU; vitamin D_3_, 2,000 IU; vitamin E, 60 IU; vitamin K_3_, 2 mg; thiamine, 2 mg; riboflavin, 5 mg; vitamin B_6_, 2 mg; vitamin B_12_, 0.028 mg; niacin, 20 mg; d-pantothenic acid, 25 mg; folic acid, 4 mg; biotin, 0.6 and 0.4 mg for gestation and lactation diets respectively. Minerals provided per kilogram of complete diet: Cu (as CuSO_4_·5H_2_O), 12 mg; Fe (as FeSO_4_·7H_2_O), 100 mg; Zn (as ZnSO_4_), 100 mg; Mn (as MnO_2_), 30 mg; I (as KI), 0.99 mg; Co (as CoSO_4_), 0.5 mg; and Se (as Na_2_SeO_3_·5H_2_O), 0.4 mg.

**Table 2:** Composition of basal diet for weaned piglets (as fed basis, %)

Items	Phase1 (d 1 to 7)	Phase 2 (d 8 to 21)	Phase 3 (d 22 to 42)
Corn (extruded)	16.02	15.22	-
Oat (extruded)	15.0	-	-
Corn	12.3	28.06	42.78
Wheat	-	10	20
Fermented soyprotein	2.3	-	-
Potato protein	1.5	-	-
Dehulled soybean meal	7.0	17	23.34
Extruded full fat soybean	-	5.0	-
Plasma protein	5.0	-	-
Dried milk powder	10	-	-
Krill powder	2.0	2.0	2.0
Cheese powder	3.5	4.0	-
Wheat bran	2.5	3.0	2.5
Yeast culture	1.0	1.0	1.0
Soybean oil	1.9	3.2	-
Animal fat	-	-	3.69
Lactose	14.0	6.0	-
Mono-calcium phosphate	0.78	1.06	0.74
Limestone	0.67	0.65	1.1
Salt	0.10	0.20	0.30
ZnO	0.25	0.25	-
Choline chloride (50%)	0.31	0.15	0.09
L-Lysine HCl (78%)	0.48	0.5	0.38
DL-Methionine (98%)	0.4	0.32	0.32
L-Threonine (98%)	0.37	0.29	0.21
L-Tryptophan (98%)	0.12	0.1	0.05
Vitamin/mineral mixture1)	2.5	2.0	1.5
Calculated composition			
Digestible energy (MJ/kg)	15.62	14.95	14.61
Metabolizable energy (MJ/kg)	14.78	14.32	13.94
Analyzed composition (%)			
Crude protein	19	18	18
Crude fat	7.0	7.0	6.0
Crude ash	4	4.5	4.8
ADF	1.7	2.4	2.6
aNDF	5.8	8.1	8.8
Lysine	1.47	1.3	1.2
Methionine	0.44	0.39	0.36
Threonine	1.03	0.87	0.8
Calcium	0.8	0.8	0.81
Phosphorus	0.55	0.55	0.5

ADF, acid detergent fiber; aNDF, neutral detergent fiber assayed with heat stable amylase.

1) Vitamins provided per kilogram of complete diet: vitamin A, 18,000 IU; vitamin D_3_, 2,500 IU (phases 1 and 2); vitamin D_3_, 1,750 IU (phase 3); vitamin E, 180 IU (phase 1 and 2); vitamin E, 80 IU (phase 3); vitamin K_3_, 4.5 mg; thiamine, 6 mg; riboflavin, 9 mg; vitamin B_6_, 7.5 mg; vitamin B_12_, 0.06 mg; niacin, 40 mg; d-pantothenic acid, 30 mg; folic acid, 2.3 mg; biotin, 0.3 mg. Minerals provided per kilogram of complete diet: Cu (as CuSO_4_·5H_2_O), 100 mg; Fe (as FeSO_4_·7H_2_O), 80 mg (phase 1 and 2) 100 mg (phase 3); Zn (as ZnSO_4_), 80 mg (phase 1 and 2), 60 mg (phase 3); Mn (as MnO_2_), 20 mg (phases 1 and 2), 40 mg (phase 3); I (as KI), 0.3 mg; Co (as CoSO_4_), 0.5 mg; and Se (as Na_2_SeO_3_·5H_2_O), 0.4 mg.

**Table 3:** Composition of basal diet for growing to finishing pigs (as fed basis, %)

Items	Growing pig (d 43 to 70)	Growing pig (d 71 to 98)	Finishing pig (d 99 to 140)
Corn	33.07	37.48	35.65
Wheat	19.0	24.0	29.0
Rice bran	2.0	2.0	2.0
Wheat bran	2.0	-	-
Pal m kernel meal	2.0	3.0	3.0
Soybean meal	3.0	3.0	3.0
Dehulled soybean meal	15.11	11.34	8.12
Rape seed meal	4.0	4.0	4.0
Sesame meal	2.0	2.0	2.0
Brown rice	5.0	5.0	5.0
Animal fat	3.79	3.26	2.89
Molasses	2.0	2.0	2.0
Bakery by product	4.0	-	-
Limestone	1.05	1.08	1.10
Mono-calcium phosphate	0.16	0.10	0.09
Salt	0.30	0.30	0.30
DL-Methionine (98%)	0.01	-	0.01
L-Threonine (98%)	0.02	0.01	0.05
L-Lysine HCl (25%)	0.50	0.49	0.79
Choline chloride (50%)	0.09	0.09	0.10
Yeast culture	0.50	0.50	0.50
Vitamin/Mineral mixture^1)^	0.40	0.35	0.40
Calculated composition			
Digestible energy (MJ/kg)	14.91	14.82	14.69
Metabolizable energy (MJ/kg)	13.73	13.65	13.61
Analyzed composition (%)			
Crude protein	17.5	16.0	15.0
Crude fat	6.7	5.9	5.5
Crude ash	4.4	4.2	4.1
ADF	4.4	4.1	4.1
aNDF	11.7	11.1	11.3
Lysine	0.99	0.88	0.86
Methionine	0.30	0.27	0.26
Threonine	0.64	0.57	0.56
Calcium	0.75	0.65	0.65
Phosphorus	0.42	0.39	0.39

ADF, acid detergent fiber; aNDF, neutral detergent fiber assayed with heat stable amylase.

1) Vitamins provided per kilogram of complete diet: vitamin A, 8,000 IU; vitamin D_3_, 1,750 IU; vitamin E, 25 IU (phase 1 and 2) 50 IU (phase 3); vitamin K_3_, 2 mg; thiamine, 3 mg; riboflavin, 4 mg; vitamin B_6_, 4 mg; vitamin B_12_, 0.03 mg; niacin, 20 mg; d-pantothenic acid, 15 mg; folic acid, 0.5 mg; biotin, 0.02 mg. Minerals provided per kilogram of complete diet: Cu (as CuSO_4_·5H_2_O), 100 mg (phase 1), 40 mg (phase 2), 5 mg (phase 3); Fe (as FeSO_4_·7H_2_O), 100 mg; Zn (as ZnSO_4_), 60 mg (phase 1), 35 mg (phases 2 and 3); Mn (as MnO_2_), 40 mg; I (as KI), 0.5 mg; Co (as CoSO_4_), 0.5 mg; and Se (as Na_2_SeO_3_·5H_2_O), 0.3 mg.

**Table 4:** Oligonucleotide primers used for a relative-quantitative real-time polymerase chain reaction analysis

Gene symbol	Description	Accession No	Primer (5′→ 3′)	

			Forward	Reverse
*MSTN*	Myostatin	NM_214435	ATGCAAGTGGAAGGAAAACC	CGTCTTTCATGGGTTTGATG
*MYOD1*	Myogenic differentiation 1	NM_001002824	CTATGATGACCCGTGTTTCG	CGTTAGTGGTCTTGCGTTTG
*MYOG*	Myogenin	NM_001012406	AGTGAATGCAGTTCCCACAG	ACTGTGATGCTGTCCACGAT
*MYF5*	Myogenic factor 5	NM_001278775	AGATCCTCAGGAATGCCATC	CATTTGGTACATCCGGACAG
*GAPDH*	Glyceraldehyde-3-phosphate dehydrogenase	NM_001206359	ACACCGAGCATCTCCTGACT	GACGAGGCAGGTCTCCCTAA

**Table 5:** Effects of supplementing the gestation and lactation diet with 25(OH)D_3_ on litter characteristics and pre-weaning growth of piglets

Items	CON^1)^	TRT^1)^	SEM	p-value
Litter size (number)				
Litter size at birth per sow	11.7	12.3	0.28	0.107
Live piglet birth per sow	11.3	12.2	0.26	0.024
Mummies	0.17	0.08	0.07	0.426
Stillborn	0.208	0.10	0.08	0.236
Fostered	11.00	11.00	-	-
Weaned	10.16	10.25	0.08	0.491
Survivability (%)	92.4	93.18	0.77	0.491
Piglet birth weight (kg)	1.07	1.09	0.01	0.250
Piglet weaning weight at day 21 (kg)	6.56	6.82	0.07	0.020
Average daily gain (g)	261	272	3.48	0.020

Values represent the means of 24 sows and their litters per treatment.

SEM, standard error of means.

1) CON per kg diet: sow, 2,000 IU D_3_; TRT per kg diet: sow, 2,000 IU D_3_+50 μg 25(OH)D_3_.

**Table 6:** Effects of supplementing 25(OH)D_3_ to sow and their progeny diets on growth performance of wean-finish pigs during different phases of growth

Items	Sow diet	CON^1)^		TRT^1)^		SEM	Time^2)^	p-value		
			
	Piglet diet	CON	TRT	CON	TRT			Sow diet effect	Piglet diet effect	Interaction effects
Body weight (kg)										
Day 1		7.23	7.25	7.3	7.4	0.38	-	0.778	0.879	0.925
Day 42		25.3	25.99	25.91	26.89	0.46	-	0.105	0.077	0.792
Day 98		70.5	72.58	71.57	73.33	0.92	-	0.333	0.045	0.868
Day 140		106	110.67	108.5	110.9	1.38	<0.0001	0.415	0.023	0.534
Day (1 to 42)										
ADG (g)		430	445.7	442.7	464	5.83	-	0.012	0.004	0.646
ADFI (g)		643	659.6	650.3	660.9	10.1	-	0.679	0.193	0.775
G/F		0.67	0.675	0.681	0.704	0.01	-	0.006	0.048	0.198
Day (43 to 98)										
ADG (g)		808	832.05	815.2	829.4	14.1	-	0.872	0.186	0.729
ADFI (g)		1,642	1,671.2	1,649	1,670	20.3	-	0.88	0.215	0.848
G/F		0.49	0.496	0.494	0.498	0.004	-	0.683	0.343	0.891
Day (99 to 140)										
ADG (g)		856	906.75	879.1	895.4	14.1	-	0.69	0.026	0.238
ADFI (g)		2,236	2,273.8	2,245	2,300	24.3	-	0.478	0.069	0.739
G/F		0.39	0.399	0.393	0.389	0.01	-	0.833	0.402	0.147
Overall (1 to 140 days)										
ADG (g)		709	738.55	722.6	739.6	9.02	<0.0001	0.425	0.016	0.493
ADFI (g)		1483	1510.3	1490	1517	12.9	<0.0001	0.579	0.044	0.974
G/F		0.48	0.488	0.483	0.486	0.004	<0.0001	0.631	0.086	0.631

Values represent means of 8 replicate pens for each sow-wean to finish treatment combination with five pigs per pen for the four sow and post weaning combinations.

ADG, average daily gain; ADFI, average daily feed intake; G/F, gain:feed ratio; SEM, standard error of means.

1) CON per kg diet: sow, 2,000 IU D_3_; nursery, 2,500 IU D_3_; growing and finishing, 1,750 IU D_3_; TRT per kg diet: sow, 2,000 IU D_3_+50 μg 25(OH)D_3_; nursery, 2,500 IU D_3_+50 μg 25(OH)D_3_; growing and finishing, 1,750 IU D_3_+50 μg 25(OH)D_3_.

2) Data were analyzed using a repeated measure mixed model with initial value at the start of the experiment as a covariate.

**Table 7:** Effects of supplementing 25(OH)D_3_ to sow and their progeny diets on blood profile of wean-finish pigs at days 42, 98, and 140

Items	Sow diet	CON^1)^		TRT^1)^		SEM	p-value		
			
	Piglet diet	CON	TRT	CON	TRT		Sow diet effect	Piglet diet effect	Interaction
Day 42									
Ca (mmol/L)		2.19	2.32	2.19	2.34	0.066	0.889	0.058	0.875
P (mmol/L)		3.07	3.19	3.18	3.28	0.114	0.382	0.354	0.914
25(OH)D_3_ (nmol/L)		92.19	95.59	90.04	97.06	1.395	0.808	0.001	0.205
IgG (g/L)		8.06	8.12	8.09	8.21	0.073	0.383	0.242	0.710
IL-1 (ng/L)		299.01	303.63	297.06	313.06	10.18	0.716	0.320	0.581
TNF-α (ng/L)		312.81	330.62	318.60	334.35	10.64	0.658	0.126	0.924
IL-6 (ng/L)		847.63	758.91	831.07	739.53	39.78	0.655	0.031	0.972
TNF-β (ng/L)		311.62	300.41	309.43	3067.67	8.30	0.762	0.441	0.574
Day 98									
Ca (mmol/L)		2.69	2.88	2.78	2.94	0.112	0.518	0.119	0.899
P (mmol/L)		2.84	3.42	2.81	3.45	0.217	1.00	0.009	0.864
25(OH)D_3_ (nmol/L)		114.9	128.74	113.75	112.34	3.61	0.267	0.005	0.424
IgG (g/L)		2.88	2.96	2.93	3.01	0.075	0.513	0.256	1.00
IL-1 (ng/L)		102.46	104.82	103.71	107.35	5.00	0.708	0.554	0.898
TNF-α (ng/L)		51.16	53.12	52.02	55.44	2.15	0.465	0.221	0.739
IL-6 (ng/L)		37.59	35.75	36.86	33.99	1.85	0.506	0.212	0.783
TNF-β (ng/L)		171.26	164.98	167.43	161.90	3.79	0.371	0.131	0.921
Day 140									
Ca (mmol/L)		2.57	3.12	2.59	2.82	0.193	0.481	0.052	0.423
P (mmol/L)		3.09	3.96	3.57	3.96	0.349	0.503	0.081	0.503
25(OH)D_3_ (nmol/L)		109.54	128.12	114.58	127.92	5.01	0.634	0.004	0.606
IgG (g/L)		2.98	3.06	2.99	3.10	0.066	0.654	0.158	0.823
IL-1 (ng/L)		98.30	103.04	97.94	109.18	3.56	0.424	0.033	0.369
TNF-α (ng/L)		48.87	50.47	50.51	51.14	1.89	0.547	0.562	0.802
IL-6 (ng/L)		38.39	35.87	36.20	34.25	1.89	0.322	0.246	0.882
TNF-β (ng/L)		177.98	169.57	169.32	167.89	3.83	0.189	0.211	0.370

Values represent means of 8 pens with 1 pig per pen.

SEM, standard error of means; IgG, immunoglobulin G; IL-1, interleukin-1; TNF-α, tumor necrosis factor-alpha; IL-6, interleukin-6; TNF-β, tumor necrosis factor beta.

1) CON per kg diet: sow, 2,000 IU D_3_; nursery, 2,500 IU D_3_; growing and finishing, 1,750 IU D_3_; TRT per kg diet: sow, 2,000 IU D_3_+50 μg 25(OH)D_3_; nursery, 2,500 IU D_3_+50 μg 25(OH)D_3_; growing and finishing, 1,750 IU D_3_+50 μg 25(OH)D_3_.

**Table 8:** Effects of supplementing 25(OH)D_3_ to sow and their progeny diets on backfat thickness and meat quality of wean-finish pigs

Items	Sow diet	CON^1)^		TRT^1)^		SEM	p-value		
			
	Piglet diet	CON	TRT	CON	TRT		Sow diet effect	Piglet diet effect	Interaction
Backfat thickness (mm)									
Day 70		7.0	7.31	7.56	7.62	0.22	0.058	0.403	0.576
Day 98		11.9	12.03	11.81	12.37	0.23	0.584	0.139	0.340
Day 140		20.75	20.8	21.2	21.3	0.25	0.074	0.625	1.00
Meat color									
L*		48.42	49.68	48.79	48.6	0.85	0.679	0.533	0.401
a*		14.13	14.76	14.8	14.71	0.31	0.322	0.395	0.263
b*		3.9	3.83	3.87	3.87	0.07	0.980	0.641	0.690
Sensory evaluation									
Color		3.29	3.32	3.32	3.42	0.10	0.547	0.547	0.717
Marbling		2.78	2.7	2.7	2.65	0.05	0.303	0.303	0.795
Firmness		2.73	2.7	2.64	2.71	0.06	0.526	0.703	0.377
Cooking loss (%)		34.32	33.47	33.57	32.63	0.59	0.190	0.140	0.937
Drip loss (%)									
d 1		6.39	6.59	6.41	6.29	0.58	0.808	0.948	0.786
d 3		12.32	12.05	11.9	11.81	0.52	0.538	0.734	0.869
d 5		18.88	16.79	17.43	16.09	0.57	0.071	0.006	0.520
d 7		24.43	22.55	23.13	22.14	0.34	0.019	<.001	0.210
pH_24 h_		5.48	5.38	5.54	5.43	0.03	0.057	<.001	0.806
*Longissimus* muscle area (cm^2^)		59.15	60.93	61.11	61.68	1.18	0.262	0.330	0.611
Water holding capacity (%)		45.05	46.36	45.25	47.34	0.56	0.298	0.005	0.485

Values represent means of 8 replicate pens with 1 pig per pen for meat quality.

Values represent means of 8 replicate pens for each sow-wean to finish treatment combination with five pigs per pen for the four sow and post weaning combinations for backfat thickness.

SEM, standard error of means.

1) CON per kg diet: sow, 2,000 IU D_3_; nursery, 2,500 IU D_3_; growing and finishing, 1,750 IU D_3_; TRT per kg diet: sow, 2,000 IU D_3_+50 μg 25(OH)D_3_; nursery, 2,500 IU D_3_+50 μg 25(OH)D_3_; growing and finishing, 1,750 IU D_3_+50 μg 25(OH)D_3_.

**Table 9:** Effects of supplementing 25(OH)D_3_ to sow and their progeny diets on relative mRNA expression of muscle genes in wean-finish pigs at day 140

Items	Sow diet	CON^1)^		TRT^1)^		SEM	p-value		
			
	Piglet diet	CON	TRT	CON	TRT		Sow diet effect	Piglet diet effect	Interaction
*MSTN*		1.121	1.081	1.035	0.934	0.02	<0.0001	0.006	0.208
*MYOD*		1.066	1.069	1.112	1.169	0.03	0.008	0.259	0.305
*MYOG*		1.071	1.093	1.082	1.086	0.02	0.926	0.608	0.709
*MYF5*		1.005	1.087	1.103	1.163	0.03	0.007	0.024	0.717

Values represent means of 8 pens with 1 pig per pen.

SEM, standard error of means; *MSTN*, myostatin; *MYOD*, myogenic differentiation; *MYOG*, myogenin; *MYF5*, myogenic factor 5.

1) CON per kg diet: sow, 2,000 IU D_3_; nursery, 2,500 IU D_3_; growing and finishing, 1,750 IU D_3_; TRT per kg diet: sow, 2,000 IU D_3_+50 μg 25(OH)D_3_; nursery, 2,500 IU D_3_+50 μg 25(OH)D_3_; growing and finishing, 1,750 IU D_3_+50 μg 25(OH)D_3_.
